# Response to Daly-Smith et al.’s commentary on ‘The Daily Mile makes primary school children more active, less sedentary and improves their fitness and body composition: a quasi-experimental pilot study’

**DOI:** 10.1186/s12916-019-1336-3

**Published:** 2019-05-22

**Authors:** Ross A. Chesham, Josephine N. Booth, Emma L. Sweeney, Gemma C. Ryde, Trish Gorely, Naomi E. Brooks, Colin N. Moran

**Affiliations:** 1 Faculty of Health Sciences and Sport, University of Stirling, Scotland, FK9 4LA UK; 2 Institute of Education, Community and Society, Moray House School of Education, University of Edinburgh, Scotland, EH8 8AQ UK; 3 Centre for Health Sciences, School of Health, Social Care and Life Sciences, University of the Highlands and Islands, Old Perth Road, Inverness, IV2 3JH UK

**Keywords:** The Daily Mile™, Children, Primary school, Physical activity, MVPA, Sedentary, Fitness, Body composition

## Abstract

We thank Daly-Smith et al. for taking the time to read the results of our pilot research study, describing it as an important and welcome contribution. Nonetheless, the authors argue six points against our conclusion. We contend that we addressed three of these points in our original discussion and disagree with their remaining points. Overall, their Commentary adds little to the topic of research into the Daily Mile™ that we had not already raised in our discussion. Additionally, they attribute statements to us that we did not make and ignore the raising of key issues in our original article. Given this, we stand by our original peer-reviewed conclusion that introducing the Daily Mile™ to the primary school day appears to be an effective intervention for increasing levels of moderate to vigorous physical activity, reducing sedentary time, increasing physical fitness and improving body composition, and that these findings have relevance for teachers, policy-makers, public health practitioners and health researchers.

## Background

We thank Daly-Smith et al. [1] for taking the time to read the results of our pilot research study [2], describing it as an important and welcome contribution. Low physical activity and high sedentary behaviour are important issues currently on the global health policy agenda [3] (e.g. Active Scotland Outcomes Framework [4]). The Daily Mile™ is a run–walk intervention with growing popularity and global reach, now being implemented in more than 8300 schools in over 50 countries [5]. Our study was not intended or designed to compare the Daily Mile™ to classroom movement breaks or physically active learning programmes; instead, we sought to investigate the effectiveness of the Daily Mile™ in terms of the anecdotally reported physiology-related benefits [2]. Our quasi-experimental pilot study provides the first assessment of the Daily Mile™, setting the starting point for the development of an evidence base.

## Response

Daly-Smith et al. [1] make six key points in their argument against our conclusion. We contend that we made three of these points in our original discussion and provide a rebuttal to their remaining points. We comment on each one in turn below.

### Correcting for gender and age on day of testing is appropriate when accounting for potentially unequal dose–response conditions

Daly-Smith et al. [1] describe our measures of moderate to vigorous intensity physical activity (MVPA), fitness and body composition as valid and reliable; these measures are all known to change with age and to differ by gender [6]. Physical activity recommendations vary for pre-school children, school children and adults [7], with fitness and body composition having a range of age- and gender-based norms and corrections (e.g. [8–10]). As acknowledged in our original article (Discussion, Strengths and limitation section –[2], p. 10–11), it would be preferable to have simultaneously included intervention and control schools for the same length of time. However, correcting for age on day of testing and gender effectively makes these variables independent of time. Thus, our correction for age and gender is both common and necessary when studying children and accounts for any differences in the dose.

### Seasonal effects are expected to be minimal, whilst correcting for gender and age on day of testing reduces any unequal opportunity for benefits

Daly-Smith et al. [1] suggest that the between-group differences in baseline outcome measures may unduly influence the study findings and that time of year may influence the measurements. It was expected that slightly older children would have different values for some of our outcome measures at baseline; to account for this, we corrected for age and gender, as clearly stated in our article (Results section, [2], p. 6). The direction of the remaining difference in sedentary time made it less, rather than more, likely that we would find a change associated with the Daily Mile™. Although we were not able to control for daily weather patterns, we already acknowledged that, “*It would have been preferable to assess both the intervention and control schools at the same time of year to avoid any seasonal impact on physical activity. However, we believe that October and March should be similar enough to allow comparison*” ([2], p. 10). We discussed this during the review process with reference to Atkin et al. [11] (Discussion, Strengths and limitation section [2], p. 10, and see also author response in ‘Open Peer Review Reports’ link in our original article); thus, we believe that we have adequately addressed Daly-Smith et al.’s comment in our original article and during the peer-review process.

### The variability in the individual response to the exercise intervention is exactly as would be expected based on a large body of literature

Daly-Smith et al. [1] attributed a statement implying that we claimed a universal benefit of the Daily Mile™ – we made no such statement. Variability in response to an exercise intervention (indeed, any intervention) is well established. A good example of this can be seen in the HERITAGE study [12], where 481 people took part in a 20-week exercise intervention. The average increase in VO_2_ max was 393 mL min^− 1^, with a range of responses from > 1000 mL min^− 1^ to a decrease of approximately − 100 mL min^− 1^. Importantly, there were no distinct responder and non-responder groups, but instead a continuum of response size. Coupled with the facts that we did not control for pupils who took up or dropped out of sports classes and that the Daily Mile™ is a self-paced activity of only 15 min duration, then the size and spread of the changes observed were exactly as would be expected. This spread in response does not detract from the typical value of taking part in the Daily Mile™. Nevertheless, we do agree that understanding why some people have larger responses than others is highly important and should be further assessed in future studies.

### Our accelerometer sample was randomly selected across the participants and should be representative of the entire sample

As we already discussed during peer review, the smaller number of children in the accelerometer analysis was due to the intervention school’s desire to start the Daily Mile™. We only included accelerometer data that we were certain was collected before the school began the intervention (Results section [2], p. 5–6). These pupils were not selected, they were simply the first to receive accelerometers because they were the first to be available for the other physiological tests, which made them relatively well spread throughout the school (see Table S1 in [2]). As stated in our article, all children involved in the study received accelerometers; however, those that received them after the introduction of the Daily Mile™ were excluded from the analysis. Whilst sensitivity analyses may be interesting, the smaller number of participants with valid accelerometer data was not due to attrition. Furthermore, we already stated that future studies should recruit larger samples across multiple schools (Discussion and Strengths and limitations sections [2], p. 11).

### Using 60 s epochs in our accelerometer analysis allows us to directly compare our results to large international studies such as the international Children’s Accelerometry database [13]

Daly-Smith et al. [1] suggest that there are four issues with our approach to accelerometers, namely that (1) we analysed data in 60 s rather than 15 s epochs; (2) we did not analyse data in epochs of ≤15 s; (3) we did not report whether participants were given the same accelerometer model in pre- and post-intervention measurements; and (4) we included weekend days in our analysis of MVPA and sedentary time. On points 1 and 2, the review by Migueles et al. [14] cited by Daly-Smith et al. [1] demonstrates that there are mixed reports on epoch length in the literature. We certainly agree that altering epoch length could change the conclusions. Thus, we chose to use the Evenson cut points [15], as recommended for school age children [16], with 60 s epochs and 60 min of non-wear time to allow direct comparison with data from the largest collection of accelerometer measurements in children – the International Children’s Accelerometry Database [13]. To address point 3, we note that the International Children’s Accelerometry Database includes studies with variable accelerometer models. It is vital that data from different studies can be compared. Further, as stated on page 4 of our original article (Methods, Participant assessments section [2], others have compared different accelerometer models and found their use acceptable in the same study. Finally, regarding point 4, Daly-Smith et al. [1] pose a good question about differences in MVPA between days on which the Daily Mile™ is completed versus days on which it is not (i.e. weekend days) and the potential for compensation; this is why we chose to look at overall MVPA and highlight (Discussion, Strengths and limitations section [2], p. 11) that further assessment of this issue is an important question for future research.

### Assessing the effectiveness of the Daily Mile™ as interpreted by the schools is a strength of our study

Daly-Smith et al. [1] suggest that we should have confirmed the contribution of the Daily Mile™ to acute MVPA responses and outcomes using a segmented day analysis. However, our study sought to assess the veracity of some of the anecdotally reported benefits to doing the Daily Mile™, including reports of children being more willing to walk and run to places outside school. Additionally, there was potential for a compensatory decrease in MVPA at other times of the day which we wished to be able to discount. Although a segmented day analysis may provide more insight into our data and be a useful approach in future studies, our more straightforward analysis was entirely appropriate to assess the impact of the Daily Mile™ on overall MVPA. Additionally, Daly-Smith et al. [1] see our lack of a treatment fidelity measure as a weakness; however, we see it as a strength. As we state in our methods (Intervention section [2], p. 4), a leaflet produced by the originator school was given to the school implementing the Daily Mile™ without additional instructions. Therefore, the form of the Daily Mile™ that we assessed is likely to be the form closest to that being taken up and maintained by schools across the globe and thus more ecologically valid than a strict prescription. Nevertheless, we also see careful assessment of the exercise dose as an important area for future research.

## Summary and conclusion

The Daily Mile™ has become a popular intervention worldwide, with many benefits being anecdotally reported. The aim of our research was, as clearly stated, to investigate only some of these anecdotally reported physiological benefits, whilst we highlighted that many more benefits remain to be assessed. We have performed additional research into successful implementation of the Daily Mile™ [17] and on its cognitive benefits [18]. We describe our work as a quasi-experimental pilot study with a clear description of our methods and results. We had already raised the majority of the criticisms of Daly-Smith et al. [1] in our article as weaknesses and have addressed their remaining issues herein. Our article states that current studies should be replicated in more schools and countries and that additional anecdotally reported benefits should be investigated. Consequently, we stand by our original conclusion that “*introducing the Daily Mile to the primary school day appears to be an effective intervention for increasing MVPA and reducing sedentary time and it has measurable impacts on key aspects of metabolic health: body composition and physical fitness*” ([2], p. 12). We believe that these findings have relevance for teachers, policy-makers, public health practitioners and health researchers.

## Abbreviation

MVPA: moderate to vigorous intensity physical activity
